# Spinal excitability is increased in the torque-depressed isometric steady state following active muscle shortening

**DOI:** 10.1098/rsos.171101

**Published:** 2017-11-22

**Authors:** Caleb T. Sypkes, Benjamin Kozlowski, Jordan Grant, Leah R. Bent, Chris J. McNeil, Geoffrey A. Power

**Affiliations:** 1 Department of Human Health and Nutritional Sciences, College of Biological Sciences, University of Guelph, Guelph, Ontario, Canada; 2 Centre for Heart, Lung and Vascular Health, School of Health and Exercise Sciences, University of British Columbia, Kelowna, British Columbia, Canada

**Keywords:** electromyography, integrated electromyography, force depression, concentric, transcranial magnetic stimulation, cervicomedullary electrical stimulation, History-Dependence of force

## Abstract

Torque depression (TD) is the reduction in steady-state isometric torque following active muscle shortening when compared with a purely isometric contraction at the same muscle length and level of activation. The purpose of the present study was to assess spinal and supraspinal excitability in the TD state during submaximal contractions of the dorsiflexors. Eleven young (24 ± 2 yrs) males performed 16 contractions at a constant level of electromyographic activity (40% of maximum). Half of the contractions were purely isometric (8 s at an ankle angle of 100°), whereas the other half induced TD (2 s isometric at 140°, a 1 s shortening phase at 40° s^−1^ and 5 s at 100°). Motor evoked potentials (MEPs), cervicomedullary motor evoked potentials (CMEPs) and compound muscle action potentials (M-waves) were recorded from tibialis anterior during the TD steady-state and purely isometric contractions. When compared with values in the purely isometric condition, following active shortening, there was a 13% decrease in torque (*p* < 0.05), with a 10% increase in normalized CMEP amplitude (CMEP/Mmax) (*p* < 0.05) and no change in normalized MEP amplitude (MEP/CMEP) in the TD state (*p* > 0.05). These findings indicate that during voluntary contractions in the TD state, the history-dependent properties of muscle can increase spinal excitability and influence voluntary control of submaximal torque production.

## 1. Introduction

It is well established that the steady-state isometric torque following a shortening muscle action is reduced when compared with that produced during a purely isometric contraction at the same muscle length and level of activation [1]. This shortening-induced reduction in torque has been termed torque depression (TD), and as a manifestation of the history-dependent nature of muscle contraction, TD has been investigated extensively for over half a century [2]. Since its initial discovery, TD has been demonstrated to occur at every functional level of muscle, and its magnitude has been positively associated with both force and displacement during shortening [3,4]. Further, TD is associated with decreased muscle stiffness, and while it may be long lasting, it is immediately abolished upon muscle relaxation [5]. Because of these observations, the basic mechanisms underlying TD have been attributed to intrinsic contractile properties of the muscle [6,7].

TD is present during voluntary maximal [8–10] and submaximal [11] contractions in humans. Given the presence of TD during volitional contractions, neural mechanisms may also contribute to the history-dependence of torque production. The relationship between TD and voluntary control is not well understood, and is only beginning to be investigated [10–13]. Considering the intrinsic force production capacity of muscle is reduced in the isometric steady state following active shortening for a given level of activation [11,12], greater motor unit activity is required to achieve similar force levels when compared with an isometric contraction without prior shortening. Given the distinct differences in activation during shortening and lengthening contractions [14], descending and peripheral synaptic inputs delivered to the motor neuron pool almost certainly differ between contraction types. These differences seem to reflect a neuromechanical coupling response to changes in muscle force production capacity under different modes of contraction, and may apply to the shortening-induced torque reduction in TD.

In a paradigm of submaximal contractions, Rousanoglou *et al*. [11] reported TD of 21 ± 3% when electromyographic (EMG) activity was held constant, and an increase in EMG amplitude of 18 ± 3% when force was held constant pre- and post-shortening. However, because subjects in the force-clamped paradigm were instructed to maintain a constant force in the TD state, it could not be determined if the increase in EMG was due in part to history-dependent neural properties or was simply a consequence of a demand for greater neural drive to overcome the mechanical aspects of TD and achieve the target force. More recently, a similar protocol demonstrated a decrease in neuromuscular efficiency (force/EMG) following shortening during a submaximal contraction compared with purely isometric contractions, owing to decreased force production and increased activation [12]. However, because surface EMG was used as a measure of global muscle activation following shortening, it was not possible to elucidate specific changes in neuromuscular activation strategies, or to identify specific sites of modulation of neural signalling within the spinal cord or motor cortex.

Hahn *et al*. [15] investigated possible adjustments of corticospinal excitability in the history-dependent state of residual force enhancement following maximal lengthening contractions. Motor evoked potentials (MEPs) and cervicomedullary motor evoked potentials (CMEPs) were recorded to identify specific modulation of excitability in the motor cortex and spinal cord, respectively. Following lengthening, enhanced torque was associated with increased MEP and unchanged CMEP amplitudes, suggesting an increase in cortical excitability but no change in motor neuron excitability in the force-enhanced state. More recently, spinal and supraspinal excitability were investigated in the TD state following maximal shortening contractions [13]. Although the mean normalized CMEP and MEP responses did not change between TD and isometric control (ISO) states, there was a significant negative relationship between CMEP and MEP amplitudes. These recent findings provide evidence that underlying mechanisms of the history-dependence of torque during voluntary contractions may not be purely mechanical, but may additionally include some level of neuromechanical coupling. While significant, the results were collected during maximal effort contractions. Submaximal contractions more accurately represent normal, everyday movements, and may influence adaptations of neural control in a history-dependent state of torque production.

The purpose of the present study was to elucidate changes in spinal and supraspinal excitability in the TD state following active muscle shortening during a submaximal contraction. To do this, neuromuscular activation (integrated EMG; iEMG) was held constant at 40% of that during a maximum voluntary contraction, and evoked potentials originating at the cortical (i.e. MEP) and spinal (i.e. CMEP) level were used to assess neural contributions to torque generation. Despite the design to match motor neuron output (i.e. iEMG) before and after the shortening contraction, we hypothesized that in the TD state, reduced torque would be accompanied by increased spinal and cortical excitability in an attempt to ameliorate the shortening-induced reduction in torque.

## 2. Methods

### 2.1. Participants

Eleven healthy male participants with a mean ± standard deviation (s.d.) age of 24 ± 4 years, height of 183 ± 8 cm and mass of 84.5 ± 6.5 kg were recruited from the university population. None of the participants had a history of neuromuscular disease or a recent ankle joint injury. Data were collected within a single session. Participants gave written informed consent prior to testing and all procedures were approved by the human Research Ethics Board of the University of Guelph (REB: 15NV008) and conformed to the Declaration of Helsinki.

### 2.2. Experimental set-up

A HUMAC NORM dynamometer (CSMi Medical Solutions, Stoughton, MA, USA) was used for torque, angular velocity and position recordings. Each participant sat with their right hip and knee angles set at 110° and 140° (180°; straight), respectfully. Joint angles were measured using a goniometer. The right knee was immobilized with the dynamometer's leg restraint (superior) and a malleable cushion (inferior), while movement at the torso was restricted with a four-point seatbelt harness. The right foot was fixed to the dorsi/plantar flexor adaptor with one inelastic strap placed over the ankle and another at the mid-distal portion of the metatarsals. The maximum ankle dorsiflexion and plantar flexion angles were set to 100° and 140° (90°; neutral), respectively, allowing for 40° of ankle excursion.

Locations for the EMG electrodes were prepared by shaving and cleaning the skin with alcohol swabs. The active electrode was placed over the tibialis anterior approximately 7 cm inferior and 2 cm lateral to the tibial tuberosity and a reference electrode was placed over the distal tendon of the tibialis anterior, at the level of the malleoli. To record antagonist activity, the active electrode was placed on the soleus, along the midline of the leg approximately 2 cm inferior to the border of the heads of the gastrocnemii, and a reference electrode was placed on the calcaneal tendon. A single ground electrode was centred on the patella. Silver–silver chloride (Ag/AgCl) electrodes (1.5 × 1 cm: Kendall, Mansfield, MA, USA) were used for all recordings.

**Figure 1.**
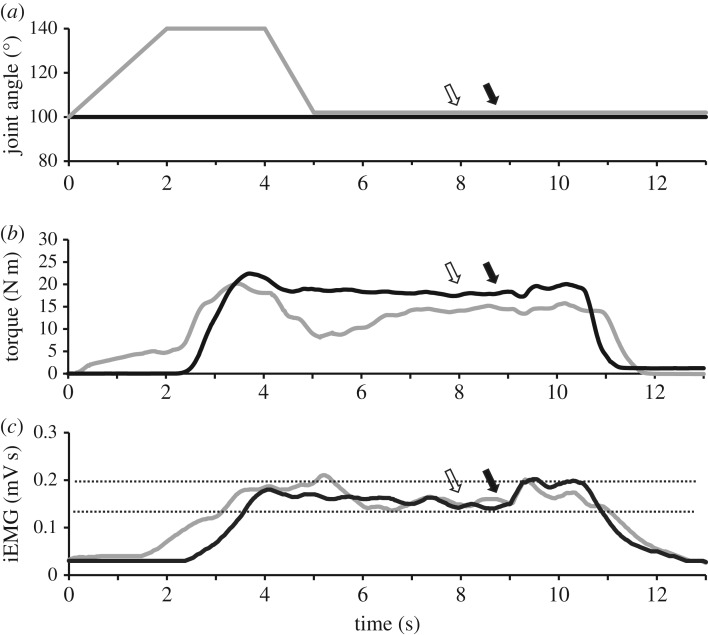
Ankle angle (*a*) as well as dorsiflexor torque (*b*) and iEMG (*c*) traces during TD (grey) and ISO (black) contractions for a representative subject. During TD trials, a contraction corresponding to 40% iEMG was initiated for 2 s at 140° plantar flexion (PF) before the dynamometer arm rotated the ankle at 40° s^−1^ to an angle of 100° PF. A maximal stimulus was delivered to the deep fibular nerve (white arrow) at the sixth second of the contraction (to elicit an Mmax), while a transcranial magnetic (TMS) or cervicomedullary stimulation (CMS) pulse (filled arrow) was administered at the seventh second (to elicit an MEP or CMEP). During isometric reference trials, the same protocol was in effect, with the exception that the ankle was fixed at an angle of 100° PF.

Raw EMG, torque, angular velocity, joint angle and stimulus trigger data were converted to digital format using a 12-bit analogue-to-digital converter (PowerLab System 16/35, ADInstruments, Bella Vista, Australia), and analysed with Labchart software (Labchart, Pro Modules 2014, v. 8). Torque and EMG data were recorded at a sampling rate of 1000 and 2000 Hz, respectively. EMG data were bandpass filtered using a digital filter (3–1000 Hz). Figure 1 depicts the joint angle, torque and iEMG traces for a single trial.

#### 2.2.1. Deep fibular and tibial nerve stimulation

**Figure 2.**
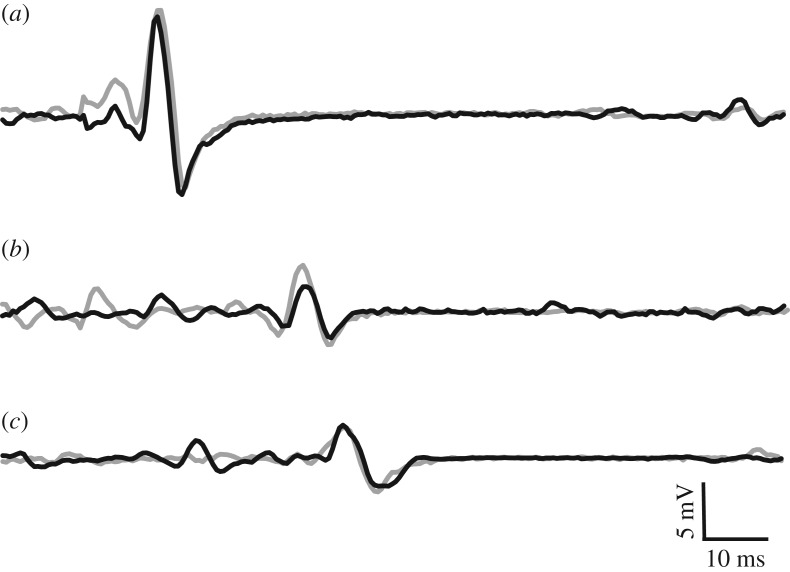
Raw data traces of an Mmax (*a*), CMEP (*b*) and MEP (*c*) recorded from the tibialis anterior following deep fibular nerve stimulation, CMS and TMS, respectively, in the TD (grey) and ISO (black) states.

To normalize voluntary EMG and evoked potentials, maximal compound muscle action potentials (M-waves) were recorded at the tibialis anterior (figure 2*a*) and soleus muscles by percutaneously stimulating the deep fibular and tibial nerves, respectively, with a standard clinical bar electrode (Empi, St Paul, MN, USA) coated in conductive gel. The deep fibular nerve was located by palpating the head of the fibula, and moving posteroinferiorly until the nerve was intercepted. The tibial nerve, innervating the plantar flexor muscles, was found by locating the distal tendon of the semitendinosus muscle and moving laterally while palpating deep into the popliteal fossa. All peripheral nerve stimuli were delivered as a single pulse from a constant current, high-voltage stimulator (model DS7AH, Digitimer, Welwyn Garden City, Hertfordshire, UK). Voltage was set to a maximum of 400 V and pulse width to 200 µs. Current was increased incrementally until a plateau was reached for the peak-to-peak amplitude of the M-wave (Mmax). To ensure consistent activation of all motor neurons throughout the experiment, the current was increased to a supramaximal level, equivalent to 110% of that required to generate Mmax.

#### 2.2.2. Maximum voluntary contraction and voluntary activation

Voluntary activation was assessed during a brief maximum voluntary contraction performed both prior to and following the experimental protocol. The interpolated twitch technique was used to evaluate voluntary activation during maximum voluntary contractions [10,16]. The torque of the superimposed twitch elicited during the plateau phase of the maximum voluntary contraction was compared to that of a resting twitch delivered 1–2 s after relaxation. The level of voluntary activation was calculated as: voluntary activation (%) = [1 − (superimposed twitch torque/resting twitch torque)] × 100%. The subjects were verbally encouraged during all maximum voluntary contractions and the torque trace was visible during each contraction [17]. All subjects were required to reach a minimum of 95% voluntary activation, and were given 5 min of rest before continuing with the experiment.

#### 2.2.3. Determining submaximal muscle activation

To determine the submaximal iEMG target, participants were instructed to perform an 8 s maximal dorsiflexion contraction at an ankle angle of 100°. The average iEMG collected between 5.5 and 6.5 s was then used to determine the 40% submaximal iEMG target. A ±5% window was calculated about this 40% target, and for all subsequent activation-controlled contractions, participants were instructed to maintain their iEMG within guidelines marking this target window.

#### 2.2.4. Cervicomedullary stimulation

Ag/AgCl electrodes (10 mm diameter; Cleartrace 1700-030, ConMed Corporation, Utica, New York, United States) were used for cervicomedullary stimulation (CMS) to generate CMEPs by passing a current across the spinal cord at the level of the mastoids. Electrodes were placed at a location approximately 2 cm superior and medial to the mastoid processes [18]. Single stimuli were presented (anode on the right side and cathode on left) with a constant current, high-voltage stimulator (DS7AH). Voltage was set to a maximum of 400 V and pulse width to 200 µs. Current was adjusted in order to produce a CMEP with an amplitude equivalent to approximately 40% of resting Mmax (figure 2*b*), while the subject performed a brief isometric contraction corresponding to 40% iEMG. This current (150–300 mA) was used for the remainder of the experiment.

#### 2.2.5. Transcranial magnetic stimulation

MEPs were elicited by transcranial magnetic stimulation (TMS) of the left motor cortex. Stimulation was delivered with a double-cone coil (110 mm) linked to two Magstim 200^2^ stimulators via a BiStim module (Magstim, Dyfed, UK). In order to determine the ideal location for coil placement, single stimuli were delivered at 20% of stimulator output while the subject performed brief control contractions at 40% iEMG. The coil was originally placed at the vertex, and was moved in 1 cm increments to the left as well as forward and backward. The placement which yielded the largest MEP was marked on the participants' scalp and used throughout the duration of testing. Stimulus intensity was adjusted until the MEP amplitude was equivalent to that of the CMEP (i.e. 40% of resting Mmax; figure 2*c*) during a brief contraction corresponding to 40% iEMG. This stimulus intensity (25–60% of stimulator output) was used for the remainder of the experiment.

### 2.3. Experimental procedures

**Figure 3.**
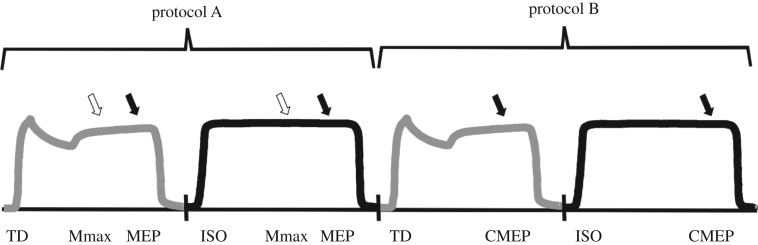
Experimental timeline. In protocol A, Mmax and MEPs were evoked in TD (grey) and ISO (black) contractions using a maximal stimulus at the deep fibular nerve (white arrow) and a TMS pulse (filled arrow), respectively. In protocol B, CMEPs were determined in TD (grey) and ISO (black) contractions using a CMS pulse (filled arrow). Protocols A and B were performed four times for a total of 16 contractions.

The experimental timeline is shown in figure 3. Each TD trial was followed by an ISO trial. Protocol A was followed by protocol B and this sequence was repeated a total of four times. Thus, four TD trials and four ISO trials were performed for each of the two protocols for a total of 16 contractions at 40% iEMG. Subjects were given visual feedback of the iEMG amplitude on the computer monitor and were verbally encouraged to match the target as closely as possible during all contractions. Three minutes of rest separated all submaximal contractions throughout the experiment.

#### 2.3.1. Protocol ‘A’: assessment of cortical and peripheral excitability

For each TD trial, the protocol consisted of a 40% iEMG contraction involving a 2 s isometric phase at an ankle angle of 140°, a 1 s isokinetic shortening phase (40° s^−1^) and a 5 s isometric phase at 100°. Although the dynamometer arm rotated the ankle at 40° s^−1^, the angular velocity was not constant throughout the entire range of motion, owing to an acceleration phase. This acceleration phase was constant across subjects (2000° s^−2^) and the phase length was minimized to limit its effects. However, its presence reduces the velocity of the ankle rotation, increasing dorsiflexion work and potentially enhancing TD. A stimulus was delivered to the deep fibular nerve at the sixth second (time point 1) and the TMS pulse was administered at the seventh second (time point 2) of the 8 s contraction. During ISO trials, an isometric 40% iEMG contraction was performed for 8 s at an angle of 100°, with stimulation of the deep fibular nerve occurring at the sixth second (time point 1) and the TMS pulse occurring at the seventh second (time point 2) of the trial.

#### 2.3.2. Protocol ‘B’: assessment of spinal excitability

The movement protocols were identical to those of protocol A, but the type of stimulation differed. CMS was delivered at the seventh second (time point 2) of each trial to correspond with the administration of TMS in protocol A.

### 2.4. Data analysis and statistics

The mean torque and root mean squared EMG (EMG_RMS_) were calculated in a 500 ms window occurring prior to each stimulus. A paired *t*-test was performed to compare the torque and EMG data between TD and ISO trials for each stimulus to validate the presence of TD at the time of stimulation. In similar studies investigating TD, non-responders are considered participants who present with no observable TD on average. There were no non-responders identified in the present study. To assess motor neuron excitability in the TD and ISO states, CMEPs were normalized to the Mmax (CMEP/Mmax) in order to control for possible changes in peripheral excitability. To assess motor cortical excitability in the TD and ISO states, MEPs were normalized to CMEPs (MEP/CMEP) to control for possible changes in subcortical excitability. The EMG_RMS_ of the resting Mmax recorded at the tibialis anterior and soleus was used to normalize the voluntary tibialis anterior and soleus EMG, respectively. The EMG_RMS_ of the soleus was used to quantify antagonist coactivation during the TD and ISO states.

In order to detect and remove outliers (30 points of 176 collected) from the dataset, MEPs and CMEPs were normalized to the subject's median evoked responses. The mean of these normalized data was calculated across participants and any response which fell more than two s.d. above or below the normalized mean was rejected.

A paired *t*-test was performed for Mmax, CMEP and MEP data between the TD and ISO states to elucidate changes in peripheral, spinal and cortical excitability in the TD state. In order to assess any effects of fatigue during the experimental protocol, a paired *t*-test was used to detect any differences in the torque produced during maximum voluntary contractions performed before and after the experiment. Descriptive data found in the text are reported as mean ±  s.d., while data presented in figures are reported as mean ± standard error of the mean (s.e.). Significant differences between the TD and ISO states were determined based on a *p*-value of less than or equal to 0.05.

## 3. Results

### 3.1. Maximum voluntary contraction and voluntary activation

Pre-trial maximum voluntary contraction torque was 35.1 ± 4.7 N m, and all participants were capable of achieving near-maximal values for voluntary activation as assessed using the interpolated twitch technique (99.4 ± 1.5%). Following the 16 contractions of the experimental protocol, maximum voluntary contraction torque and voluntary activation were reassessed. The post-trial maximum voluntary contraction torque value (33.8 ± 5.9 N m) was not statistically different from the pre-trial value (*p* > 0.05), and all participants remained capable of achieving near-maximal voluntary activation values (98.6 ± 1.6%).

### 3.2. Dorsiflexion torque and muscle activity

**Figure 4.**
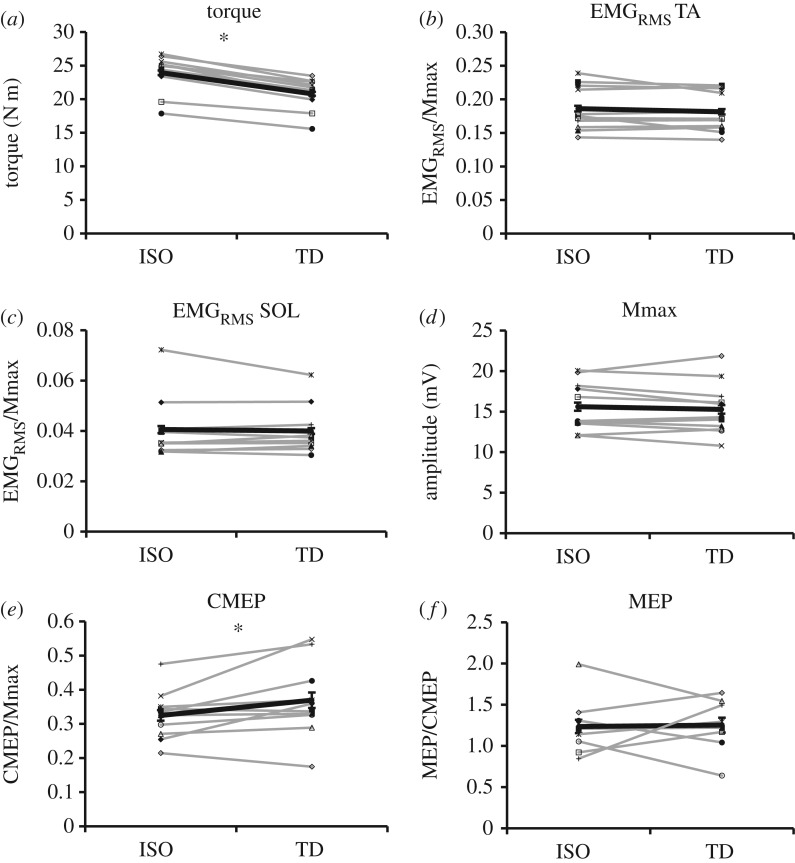
Graphs depicting the mean values for each participant (grey lines) and the group mean (black line; error bars indicate standard error of the mean) in the TD and ISO states. There was a 13.1% reduction in torque (*a*), and a 10.3% increase in normalized CMEP (*e*) in the TD state when compared with the ISO state (*p < 0.05). There was no significant difference in EMG_RMS_ collected from the tibialis anterior (*b*) or soleus (*c*), Mmax (*d*) or normalized MEP (*f*) between the two states (*p* > 0.05).

Following active shortening, steady-state isometric torque was significantly less (*p* ≤ 0.05) than that produced during the purely isometric contractions at the corresponding muscle length and level of activation (figure 4*a*), resulting in an average TD across all contractions of 13.1 ± 6.4%. Participants successfully maintained the EMG target level such that iEMG of the tibialis anterior did not differ prior to stimulation in the TD and ISO contractions (*p* > 0.05), indicating that motor neuron output was similar in both the TD and ISO states. Given the requirement to maintain iEMG, as expected, EMG_RMS_ of both tibialis anterior and soleus were not significantly different (*p* > 0.05) between TD and ISO contractions (figure 4*b*,*c*).

### 3.3. Evoked muscle responses in the torque-depressed state

There was no significant difference (*p* > 0.05) in Mmax peak-to-peak amplitude between the TD and purely ISO state (figure 4*d*). During the ISO state, the peak-to-peak amplitudes of CMEPs and MEPs were 36.1 ± 5.4% and 44.5 ± 9.8% of the resting Mmax, respectively, and were not significantly different from each other (*p* > 0.05). When normalized to Mmax (CMEP/Mmax), there was a significant (*p* ≤ 0.05) 10.3 ± 25.3% increase in CMEP amplitude during TD contractions when compared with ISO contractions (figure 4*e*). When MEPs were normalized to CMEPs (MEP/CMEP), there was no significant difference (*p* > 0.05) in the mean amplitude of MEPs between the TD and ISO contractions (figure 4*f*).

## 4. Discussion

By using TMS and CMS, the current study investigated excitability of the central nervous system in the TD state of a submaximal contraction. It was shown that following active muscle shortening, there was a significant increase in CMEP amplitude when compared with the purely isometric contractions. Therefore, in line with the hypothesis, during a submaximal contraction in the TD state, there was an increase in spinal excitability. Contrary to the hypothesis, there was no difference in MEP amplitude between contraction types. However, in some subjects, spinal and supraspinal excitability appear to be inversely related, where an increase or decrease in spinal excitability was accompanied by an opposite change at the level of the motor cortex (figure 4*e,f*). While TD is indeed an intrinsic fundamental property of skeletal muscle, these findings indicate neuromechanical coupling during voluntary contractions in the TD state, such that the history-dependent properties of muscle can influence voluntary control of force production.

### 4.1. Torque depression and antagonist coactivation

TD was observed for each subject. Further, the range of the average shortening-induced TD values for each subject (8.4–18.0%) is in line with several earlier studies which described the existence of TD in humans during voluntary contractions [9–13,19–21]. Relative to force depression in an isolated muscle preparation, it is important to acknowledge the added complexities of assessing, in humans, shortening-induced TD. Because the present study measured torque about a joint rather than the force of a single muscle, the competing influences of both agonist and antagonist muscles in the production of net joint torque must be considered. The primary agonists contributing to ankle dorsiflexion are the tibialis anterior, extensor digitorum longus, extensor hallucis longus and fibularis tertius. In the present study, tibialis anterior EMG_RMS_ was used as a measurement of global agonist activation. Participants were instructed to maintain tibialis anterior iEMG at a constant level pre- and post-shortening in order to match motor neuron output. Accordingly, agonist activation (EMG_RMS_) did not differ between TD and ISO trials. Additionally, there are seven antagonists contributing to plantar flexion. Soleus EMG_RMS_ was used as a measurement of global antagonist activation, and in keeping with previous findings [9–11,20], it was found that antagonist muscle activation did not change in the TD state when compared with ISO conditions. Importantly, while surface EMG is used to measure the output of both the central and peripheral nervous systems, the present study demonstrates that although muscle activation may appear unchanged in the TD state, neural within the central nervous system can be modified following active muscle shortening.

### 4.2. Coupling of mechanical and neural aspects of torque depression

To date, the basic mechanisms underlying TD have been attributed to the contractile components within the muscle. The most supported theory proposes that TD is related to a stress-induced inhibition of cross-bridge attachment in the actin–myosin overlap zone that is newly formed upon shortening [6,7,9]. When the muscle is activated, there is a stress-induced distortion of the thin actin filaments in the I-band region. Following active shortening, upon entering the isometric steady state, these deformed actin filaments form fewer cross-bridge attachments with thick myosin filaments, reducing muscle force production when compared with a purely isometric contraction [6]. Further, in addition to reduced cross-bridge attachment in the newly formed overlap zone, the TD state is associated with reduced force production per cross-bridge [7].

The mechanical properties underlying TD are well established. What is less certain in previous investigations is the influence of TD on voluntary neuromuscular control. While investigations have only begun recently, there have already been several findings that seem to indicate the involvement of neural mechanisms in the history-dependence of force. It has been shown that greater levels of motor unit activity are required to produce the same force in the TD state when compared with the ISO state at the same muscle length [11,12]. While these studies demonstrated an altered relationship between force production and muscle activation, they could not identify at what level(s) of the nervous system changes occurred. More recently, it was shown that excitation or inhibition at the spinal level was accompanied by an opposite change at the motor cortex in the TD state following maximal shortening contractions [13]. This counterbalancing effect provides insight into how modulations in spinal or supraspinal excitability may occur following active muscle shortening even if motor neuron output, as measured by surface EMG, remains constant [13]. Central nervous system excitability was also altered in the isometric steady state following maximal lengthening contractions [15]. It was found that the force-enhanced state was associated with increased MEP amplitude and unchanged CMEP amplitude, suggesting an increase in cortical excitability following active lengthening. Collectively, these findings indicate that voluntary control of force during maximal efforts in the history-dependent state of residual force enhancement and TD is associated with changes in excitability of the corticospinal tract.

The current study investigated neuromechanical coupling following submaximal contractions in the TD state. It was found that, following active muscle shortening, there was an increase in CMEP amplitude of approximately 10%. As seen in figure 4*e*, this was a robust finding, with only two participants showing a modest decrease. This indicates that in the TD state, there is an increase in spinal excitability, and further supports the notion of neuromechanical coupling as a phenomenon involved in the history-dependent nature of muscle contraction. There was no observed difference in MEP amplitude between the TD and ISO states, and the variability in data between subjects was considerably larger than CMEP amplitude (figure 4*f*). However, in some subjects, changes in MEP and CMEP amplitudes appear to be negatively related to one another, meaning that an increase or decrease in CMEP amplitude could have been accompanied by an opposite change in MEP amplitude. While this was not observed for all subjects, it is possible that during submaximal contractions, similar to maximal contractions [13], the excitatory changes at the spinal level were counterbalanced by reduced excitability at the level of the motor cortex. Future investigations should more closely examine this relationship.

### 4.3. Possible sensorimotor contributions to altered excitability in the torque-depressed state

The cause of increased spinal excitability in the present study is unknown, but it may be related to the function of sensory afferent feedback mechanisms located in the periphery. The Golgi tendon organ (GTO) is anatomically located in-series with the muscle and aponeurosis at the muscle–tendon junction, and is a mechanoreceptor responsible for monitoring muscle tension and providing inhibitory sensory feedback via Ib afferents to the agonist motor neuron pool [22]. Firing of Ib afferents is modulated in a tension-dependent manner [23], and signals whole-muscle force rather than internal forces related to individual motor units [24]. In the present study, torque amplitude was approximately 13% lower in the TD state when compared with the ISO state, so a reduction in Ib afferent firing could have contributed to the larger CMEPs (i.e. increased spinal excitability) observed in TD.

In addition to providing inhibitory feedback to the agonist motor neuron pool at the spinal level, Ib afferents also cause presynaptic inhibition of afferents via primary afferent depolarization [22]. Primary afferent depolarization may be used to target other Ib afferents as a negative feedback mechanism [25], but it may also be directed towards Ia afferents [26]. Presynaptic inhibition may therefore control the balance of excitatory and inhibitory feedback to the central nervous system, which is critical for voluntary force control [22] and could be altered in the history-dependent state. Further, Ib afferents relay information from GTOs to the cerebellum and cerebral cortex via the dorsal and ventral spinocerebellar tracts for higher-level proprioceptive processing [27]. The precise outcome of this input is unknown, but along with associated messages from skin, joints and other muscle receptors, it may be used for conscious sensation, intentional force adjustments and the voluntary control of movement [22]. Reduced muscle force production capacity in the TD state—while maintaining similar levels of activation—may perhaps alter the feedback delivered by these afferents, and could modify the control of force production via the central nervous system. Thus, altered afferent feedback provides an exciting area for further research into the effects of the history-dependence of force on nervous system activity and the voluntary control of force.

## 5. Conclusion

The present study investigated alterations to the excitability of the central nervous system in the torque-depressed state as indicated by changes in MEP and CMEP amplitude. Using a paradigm of maintained motor neuron output (i.e. matching of agonist integrated EMG), it was shown that following active shortening, steady-state isometric torque was significantly less than the torque produced during the purely isometric contractions. Further, it was shown that during contractions in the torque-depressed state, there was a significant increase in CMEP amplitude when compared with contractions in the isometric state, indicating increased spinal excitability in the steady state following active shortening. Overall, while TD is indeed an intrinsic fundamental property of skeletal muscle, these findings indicate that, during voluntary contractions in the torque-depressed state, the history-dependent properties of muscle can influence voluntary control of submaximal torque production.

## Supplementary Material

Supporting data - Spinal excitability is increased in the torque-depressed isometric steady-state following active muscle shortening

## Data accessibility

Individual values of all supporting data are accessible as electronic supplementary material.
